# 

**Published:** 1912-04-15

**Authors:** 


### ANNOUNCEMENTS.

Death of President Gilbert. Mr. William H. Gilbert, President of the S. S. White Dental Manufacturing Co., died, March 16, 1912, at his home, in Philadelphia. Mr. Gilbert was for many years the general superintendent of this great concern, and was elected president after the death of Mr. Lewis. He practically grew up with the business and was an important factor in placing this great corporation at the forefront of all the dental supply establishments of the world. He was a man of strong character and great ability and his loss to the corporation will be great.

Miller Memorial Fund. To the Dental Profession of America.

The committee appointed at the December '09 meeting of the Ohio State Dental Society, to raise funds to establish an American Memorial to perpetuate the memory of the late Dr. W. D. Miller, have through the co-operation of the Honorary Committees of the several states collected funds amounting to $3812.50, with an additional $450.00, subscribed but not paid in at this writing. The amount asked for from the several states was pro-rated according to the membership of the state societies, several states have over subscribed the amount called for, others partially, while ten have failed to subscribe anything.

The proposed memorial will be a monument to consist of a life size bronze of Dr. Miller mounted upon a granite base, with appropriate tablets, the cost of which will approximate $8000, and be a lasting credit to the profession. It is the desire of the committee to have a tablet stating that funds were received from representatives of the dental profession in every state of the Union, and to this end we are soliciting funds, whether they be personal or society contributions. Ohio, Dr. Miller's native state, has contributed $1400 to the fund, which amount, through personal subscriptions and component societies will be increased to about $2000.

We are asking for a favorable consideration of the matter, by the various state societies during the coming meetings, and hope the wide spread appreciation of Dr. Miller's work for our profession, will enable our committee to take steps toward the construction of this tribute to his memory at an early date.

Dr. Weston A. Price, 10406 Euclid Ave., Cleveland, Ohio, has been selected treasurer of the fund, and to him all subscriptions should be made payable.

Yours very truly,

Edward C. Mills, *Chairman*,
151 E. Broad St., Columbus, O.
J. R. Callahan,
25 Garfield Place, Cincinnati, O.
S. D. Ruggles,
Portsmouth, O.

COLORADO DENTAL ASSOCIATION. The 26th Annual Meeting of the Colorado State Dental Association will be held at Colorado Springs, June 20, 21 and 22, 1912. Dr. A. W. Starbuck, Colorado College of Dental Surgery, Denver, Colo., will have complete charge of clinics. He will gladly furnish any information relative to the same. Exhibitors desiring space please address the secretary.

All ethical practitioners are cordially invited to attend this meeting. Any information desired will be cheerfully furnished by the secretary.

H. F. HOFFMAN, *President*,
324 Metropolitan Bldg.,
Denver, Colo.
CHAS. A. MONROE, *Secretary*,
Boulder, Colo.

---

INSTITUTE OF DENTAL PEDAGOGICS. At the last meeting of the Institute of Dental Pedagogics, the Executive Board decided to hold the next annual meeting in Pittsburgh, January 28, 29 and 30, 1913. The following officers were elected for the ensuing year.

DR. H. EDMUND FRIESELL, *President.*
1206 Highland Bldg.,
Pittsburgh, Pa.
DR. D. H. SQUIRE, *Vice-President.*
Buffalo, N. Y.
DR. FRED W. GETHRO, *Secretary-Treas.*
917 Marshall Field Bldg.,
Chicago, Ill.

---

WASHINGTON UNIVERSITY DENTAL SCHOOL ALUMNI ASSOCIATION. The annual clinic and reunion of graduates will be held under the auspices of the Alumni Association of Washington University Dental School, May 6th and 7th, 1912, at the College Building, Twenty-ninth and Locust Streets, St. Louis, Mo. (The former dates of April 22nd and 23rd, first decided upon for the meeting, were changed to enable fellow alumnus, Dr. G. V. Black of Chicago, to attend as guest of honor.)

This meeting promises to be unusually interesting, both in the way of papers and clinics, and all graduates should make a special effort to be present. All ethical dentists are invited to attend.

R. A. Harris,
E. A. Woelk,
F. E. Meyer,
*Publicity Committee.*

Annual Meeting of the N. J. State Dental Society July 17, 18 and 19, 1912. The Committee in charge announce with pleasure that contracts have been signed for the Forty-second Annual Convention of the New Jersey State Dental Society, to be held in the new and magnificent one million dollar Fireproof Hotel "Cape May," Cape May, N. J.

This hotel is situated on the extreme lower corner of the New Jersey Coast, swept by the Atlantic Ocean and Delaware Bay, with miles of board walk, a sloping hard, firm beach that will accommodate six autos traveling abreast, fine bath houses and garages; the hotel has broad corridors, is thorough ly fireproof, one hundred and fifty baths with hot and cold sea water, and is unsurpassed for elegance, comfort, and cuisine; its facilities for meeting room are perfect, fine light for clinics, and the space for dental and medical exhibitions is in abundance. A direct current, electric plant is run by the hotel, and the city power is the alternating; every inducement and space will be given exhibitors. Lithographed floor plans can be obtained from the chairman of the Exhibit Committee, Dr. Moore Stevens, of 1503 Pacific Avenue,

Atlantic City, to whom application for space can be made.

The Chairman of the Clinic Committee, Dr. M. R. Brinkman, of Hackensack, already has quite a number of prominent men booked, and will try to make room for all who desire to give chair or table clinics.

The Chairman of the Essay Committee, Dr. Wentworth Holmes, 472 Broad Street, Newark, N. J., has secured from five well-known men papers of great interest and expects others.

The hotel rates will be American plan—$3.50 per day each for two persons in a room, $4.00 per day, one person in a room. Most of the rooms have twin beds. Other hotel rates in May.

Write at once and reserve your rooms; do not delay. The Pennsylvania and Reading Railroads have finely equipped cars and rapid service.

The one million five hundred thousand land-locked harbor has at present a depth of forty feet. Negotiations are under way to bring the public by the all-water route from New York, Boston, Philadelphia, Baltimore, Washington and other cities.

CHARLES A. MEEKER, D.D.S., *Sec.*

---

MICHIGAN STATE BOARD OF EXAMINERS. The next regular meeting of the Michigan State Board of Dental Examiners will be held at the Dental College, Ann Arbor commencing Monday June 17th, at 8 A. M., and continuing through the 22nd. For application blanks and full particulars address:

F. E. SHARP, *Secretary,*
Port Huron, Mich.

---

INDIANA STATE DENTAL ASSOCIATION, will hold its next meeting in Indianapolis, May 21, 22 and 23, 1912, at the Claypool Hotel. It is the plan of the Officers to secure 1000 members for this year, and to give them the best meeting ever held.

O. U. King, *Secretary*,
Huntington, Ind.

New Edition of Irwin's Dental Laws and Dental License Requirements. Since the last publication, entitled "Dental Laws, Condensed, or, Dental License Requirements," reports have been received from the following countries.:

| | | |
|---|---|---|
| Alaska, | Costa Rica, | Malta, |
| Algeria, | Cuba, | Mexico, |
| Argentine Rep., | Denmark, | New Providence I., |
| Arabia, | Dominican Rep. | Netherlands, |
| Australia, | Egypt, | Portugal, |
| Austria, | England, | Russia, |
| Barbadoes, | France, | Roumania, |
| Belgium, | Germany, | Scotland, |
| Bermuda, | Hawaii, | Servia, |
| Bulgaria, | Holland, | Sweden, |
| British E. Africa, | Hungary, | Switzerland, |
| Canada, | India, | U. S. Boards, |
| Cape Colony, | Italy, | Zanzibar, |
| Ceylon, | Jamaica, | |

One Hundred and Forty-four authorities, officially representing as many governments in the world, have furnished information in regard to the credentials a dentist must possess in order to practice dentistry in the respective countries. One hundred of this number have been heard from within the last sixty days.

Alphonso Irwin, D.D.S.
425 Cooper St.,
Camden, N. J.

KENTUCKY STATE DENTAL ASSOCIATION, will hold its meeting this year in Louisville, May 27, 28 and 29, several essayists and clinicians from other states will help to make the meeting interesting and profitable to all who will attend.

W. M. RANDALL, *Secretary.*

---

ILLINOIS STATE DENTAL SOCIETY, will hold its annual meeting this year in Springfield, May 14th to 17th.

---

TENNESSEE STATE DENTAL ASSOCIATION. The forty-fifth annual meeting of the Tennessee State Dental Association will be held at Memphis, Tenn., in the Business Men's Club Rooms, June 6, 7 and 8, 1912. The Business Men's Club extends the courtesies of the club to all the visiting dentists, and the association invites all ethical dentists to attend.

CHAS. A. TAVEL, *Pres.*
J. L. MANIRE, *Sec'y.*

---

NEW REMEDY FOR BURNS. Editor DENTAL REGISTER.—A few days ago I accidently turned the flame of my blow-pipe on to my left hand completely singeing it and causing considerable pain. In hurriedly looking around for a remedy my eye rested on my bottle of "Ideal Soldering Fluid" put up by The S. A. Crocker Co. This I applied with a very happy result, instantly relieving the pain which did not return. I do not know the formula of this fluid, but this experience may help others who keep it on hand. I think it works better and is in a more convenient form than bi-carbonate of soda with which I have had some experience for the same purpose.

Fraternally yours,
H. H. WILSON,
Phoenix, Arizona.

